# Supplementary material for: The interactome of intact mitochondria by cross-linking mass spectrometry provides evidence for coexisting respiratory supercomplexes
Source: Mol Cell Proteomics. 2017 Dec 8;17(2):216–32. doi: 10.1074/mcp.RA117.000470 (PMC5795388; doi:10.1074/mcp.RA117.000470)
Supplement: Supplemental Data [file supp_RA117.000470_133922_0_supp_23978_fzffwf.zip › spectra_annotation/mito_spectra_annotation/456-1-10-1-13-1.pdf]

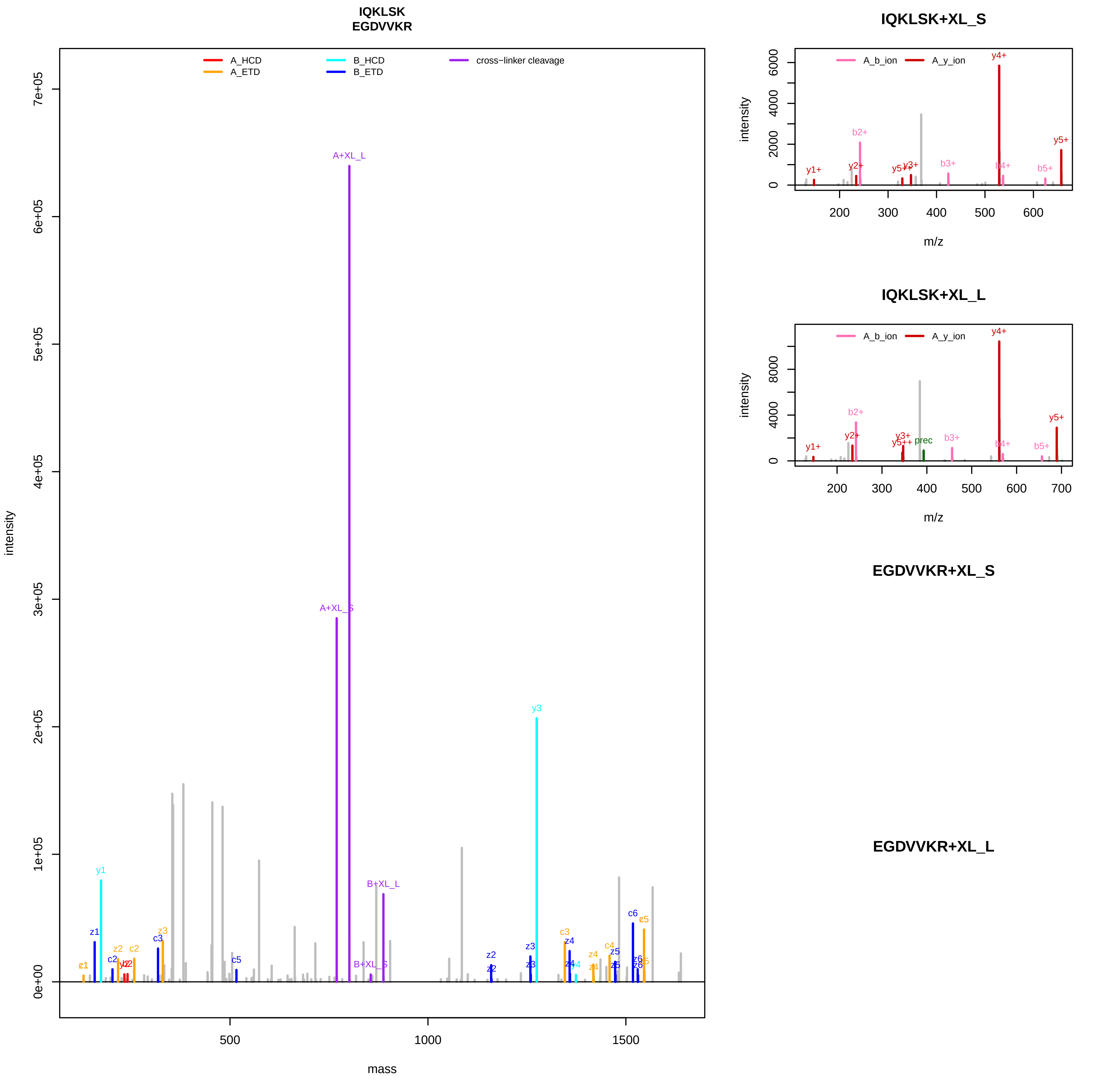
IQKLSK
EGDVVKR
A_HCD
A_ETD
B_HCD
B_ETD
cross−linker cleavage
A+XL_L
A+XL_S
B+XL_L
B+XL_S
y1
y3
z1
c2
z2
c3
z3
c5
c6
c4
z4
z5
z6
c1
intensity
mass
0e+00
1e+05
2e+05
3e+05
4e+05
5e+05
6e+05
7e+05
500
1000
1500
IQKLSK+XL_S
A_b_ion
A_y_ion
y1+
y2+
y3+
y4+
y5+
y5++
b2+
b3+
b4+
b5+
intensity
m/z
0
2000
4000
6000
200
300
400
500
600
IQKLSK+XL_L
A_b_ion
A_y_ion
y1+
y2+
y3+
y4+
y5+
y5++
prec
b2+
b3+
b4+
b5+
intensity
m/z
0
4000
8000
200
300
400
500
600
700
EGDVVKR+XL_S
EGDVVKR+XL_L
